# Nodal staging score for adequacy of nodal staging in cervical cancer

**DOI:** 10.1016/j.heliyon.2024.e26116

**Published:** 2024-02-08

**Authors:** Rui Jiang, Xiaoqi Li, Siyu Cao, Yong Wu, Wei Zhang, Yan Huang

**Affiliations:** a Department of Radiation Oncology, Fudan University Shanghai Cancer Center, Shanghai, 200032, China; b Department of Gynecologic Oncology, Fudan University Shanghai Cancer Center, Shanghai, 200032, China

**Keywords:** Cervical cancer, Beta-bimonial distribution, Nodal staging score, Prognostic analysis

## Abstract

**Background:**

Cervical cancer remains the fourth most common female malignancy with increasing newly cases around the world. It is of clinical value to precisely evaluate whether false negative nodal existed and develop a nodal staging model in cervical cancer.

**Materials and methods:**

Clinical data of cervical cancer patients was retrieved from the Surveillance, Epidemiology, and End Results database. Probability of missing nodal disease and nodal staging score (NSS) was computed to assess the nodal status of each individual.Prognostic value of NSS was assessed.

**Results:**

A total of 9056 individuals were in this study, with 5115 squamous cell carcinoma, 2791 adenocarcinoma, 512 adenosquamous carcinoma, and 638 other type individuals. A beta-binomial model was used to compute the probability of nodal disease in four histological types, respectively. False negative probability drastically decreased as more nodes examined. To reach 0.05 of false negative probability, it required at least 17 lymph nodes in squamous cell carcinoma patients,18 in adenocarcinoma, 12 in adenosquamous carcinoma patients and 14 in other types. To reach 0.95 of NSS, it took 10 lymph nodes in squamous cell carcinoma, 6 in adenocarcinoma, 10 in adenosquamous carcinoma and 7 in other types. Significant prognostic values of NSS quartiles subsets were found in all four histological sets.

**Conclusion:**

NSS tool enables adequate nodal staging of cervical cancer with significant prognostic value. Exact number of lymph nodes required for surgery in cervical cancer is specified based on histologic type.

## 1 Introduction

Cervical cancer remains one of the commonest malignancies in women with approximately 604,127 newly occurred cases and 341,831 deaths around the global in 2020 [1,2]. It is the fourth most common female malignancy with increasing health challenges in low-and middle-income countries [2]. Histological types of cervical cancer can be mainly categorized into four types, including squamous cell carcinoma, adenocarcinoma, adenosquamous carcinoma and other types [3]. Squamous cell carcinoma accounts for approximately 70% of all cervical cancer cases, andadenocarcinoma type accounting for nearly 25% of the rest [2]. Different histological types of cervical cancer may be useful to predict the treatment efficiency or survival outcome [4,5].

Surgical treatment including lymphadenectomy for cervical cancer is still a primary choice for patients below stage IIB [2]. Although the risk of lymph node metastasis is rare in stage IA1, it has been considerably increased up to 8% for stage IA2 even with radical hysterectomy [2]. Although lymph node dissection area of pelvic lymphadenectomy, including the obturator, internal, external and common iliac nodes, has been clear, the specific number of dissected total lymph nodes as well as methods to evaluate the adequacy of nodal status remain largely sparse. In fact, National Comprehensive Cancer Network guideline recommended that if negative results of pelvic lymph nodes were confirmed, radiation or not was considered adherent care [6]. Nonetheless, if the dissected pelvic lymph nodes were positive, pelvic radiation with or without brachytherapy is required [6]. Therefore, it is of paramount significance to precisely evaluate whether false negative nodal existed in cervical cancer.

To statistically assess the possibility of potential hidden lymph nodes with metastasis status, a lymph node based nodal stating score (NSS) has been developed previously in several types of malignancies, including gastric cancer, bladder cancer and epithelial ovarian cancer [[7], [8], [9]]. The NSS model is established in this study to assess the possibility of missing positive lymph nodes in a given cervical cancer case. Of note, the correlation between histological types of cervical cancer and lymph nodes staging is largely unexplored. Hereby, this study established a NSS model based on a population-based Surveillance, Epidemiology and End Results (SEER) database and various histologic types.

## 2 Materials and methods

**Fig. 1:**
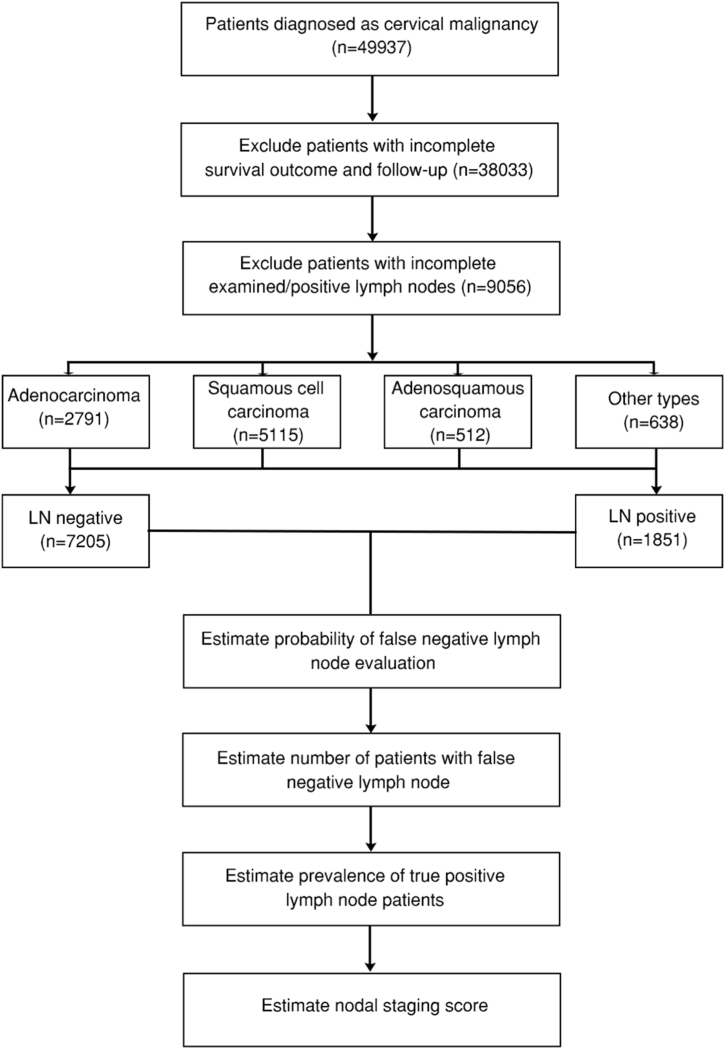
Consort diagram of cervical cancer patients retried from the Surveillance, Epidemiology, and End Results (SEER) database and input for modeling process. LN: lymph node. The selected 9056 cervical cancer patients were divided into four histological sets, including adenocarcinoma (n = 2791), squamous cell carcinoma (n = 5115), adenosquamous carcinoma (n = 512) and other types (n = 638). Next, all of them were also categorized into lymph node positive (n = 7205) and negative (n = 1851) sets, and input for the establishment of model.

All the cervical cancer patients with complete survival outcome, follow-up, examined and positive lymph nodes were retrieved from the SEER database for further modeling (Fig. 1). Clinical variables included TNM stage, ICD-0-3 SEER histology, lymph node examined/positive, tumor size, age, distant metastasis (bone/brain/liver/lung) and race. All the listed ICD-0-3 histological classification of each individual was reassigned into four major histological sets, squamous cell carcinoma, adenocarcinoma, adenosquamous carcinoma and other types.

The original algorithm of this study was mainly based on the study from Gönen et al. and Robinson et al. [10,11]. The establishment of this model was based on three steps and three assumptions:

Step I Compute the probability of missing nodal disease (P(FN_n_)) as a function of lymph nodes examined, in each histological set. A β-binomial model was used in this process (Equation (1)). β() meant the βfunction. αandβ,were defined as given parameters during the βfunction calculation, while n indicate the number of examined lymph nodes.

(1) $$P\left({FN}_{n}\right)=\frac{\beta \left(\alpha ,\beta +n\;\right)}{\beta \left(\alpha ,\beta \right)}$$

Step II Compute the true number of positive lymph nodes (Equation (2)). (*No.FN*_*n*_) indicated the number of patients with false negative lymph nodes. (*No. TP*_*n*_) indicated the number of patients with true positive lymph nodes.

(2) $$No.{FN}_{n}=\frac{P\left({FN}_{n}\right)*\left({No.TP}_{n}\right)}{1-P\left({FN}_{n}\right)}$$

Step III Compute the true prevalence of positive lymph nodes in each histological set (Equation (3)).

(3) $${\mathrm{Pr}}_{T}=\frac{\sum_{n}\left(No.{FN}_{n}+{No.TP}_{n}\right)}{\sum_{n}\left(No.{FN}_{n}+{No.TP}_{n}+{No.TN}_{n}\;\right)}$$

### 2.1 Step IV compute the NSS (Equation (4))

(4) $${NSS}_{n}=\frac{1-{\mathrm{Pr}}_{T}}{1-{\mathrm{Pr}}_{T}+{\mathrm{Pr}}_{T}*P\left({FN}_{n}\right)}$$

Assumption I No false positive reports of pathological lymph nodes in this study occurred.

Assumption II All the examined/positive lymph nodes in this study are all changeable.

Assumption III Sensitivity stays the same between true positive and false negative cases.

## 3 Results

A total of 9056 individuals were included in this study, with 5115 squamous cell carcinoma, 2791 adenocarcinoma, 512 adenosquamous carcinoma, and 638 other type individuals (Table 1, Fig. 1).

**Table 1:** Characterization of included four types of cervical cancer patients in this study divided by histological features (Squamous cell carcinoma, adenocarcinoma, adenosquamous carcinoma, other types).

	Level	ADENOCARCINOMA	ADENOSQUAMOUS CARCINOMA	OTHER TYPES	SQUAMOUS CELL CARCINOMA	p
n		2791	512	638	5115	
Histology (%)	ADENOCARCINOMA	2791 (100.0)	0 (0.0)	0 (0.0)	0 (0.0)	<0.001
	ADENOSQUAMOUS CARCINOMA	0 (0.0)	512 (100.0)	0 (0.0)	0 (0.0)	
	OTHER TYPES	0 (0.0)	0 (0.0)	638 (100.0)	0 (0.0)	
	SQUAMOUS CELL CARCINOMA	0 (0.0)	0 (0.0)	0 (0.0)	5115 (100.0)	
LN_positive (%)	Negative	2393 (85.7)	364 (71.1)	508 (79.6)	3940 (77.0)	<0.001
	Positive	398 (14.3)	148 (28.9)	130 (20.4)	1175 (23.0)	
T_stage (%)	T0	0 (0.0)	0 (0.0)	0 (0.0)	1 (0.0)	0.011
	T1	124 (4.4)	10 (2.0)	11 (1.7)	146 (2.9)	
	T2	9 (0.3)	2 (0.4)	1 (0.2)	21 (0.4)	
	T3	7 (0.3)	2 (0.4)	2 (0.3)	5 (0.1)	
	T4	2 (0.1)	0 (0.0)	0 (0.0)	4 (0.1)	
	TX	2649 (94.9)	498 (97.3)	624 (97.8)	4938 (96.5)	
N_stage (%)	N0	125 (4.5)	11 (2.1)	11 (1.7)	143 (2.8)	0.001
	N1	18 (0.6)	3 (0.6)	4 (0.6)	36 (0.7)	
	NX	2648 (94.9)	498 (97.3)	623 (97.6)	4936 (96.5)	
M_stage (%)	M0	139 (5.0)	14 (2.7)	15 (2.4)	173 (3.4)	0.002
	M1	5 (0.2)	0 (0.0)	0 (0.0)	6 (0.1)	
	MX	2647 (94.8)	498 (97.3)	623 (97.6)	4936 (96.5)	
Age (%)	<30	163 (5.8)	43 (8.4)	45 (7.1)	399 (7.8)	<0.001
	≥70	155 (5.6)	29 (5.7)	52 (8.2)	261 (5.1)	
	30≤ <50	1714 (61.4)	302 (59.0)	319 (50.0)	2959 (57.8)	
	50≤ <70	759 (27.2)	138 (27.0)	222 (34.8)	1496 (29.2)	
Tumor size (%)	<2 cm	114 (4.1)	3 (0.6)	8 (1.3)	122 (2.4)	<0.001
	2 cm<= <4 cm	127 (4.6)	19 (3.7)	18 (2.8)	186 (3.6)	
	≥4 cm	123 (4.4)	7 (1.4)	22 (3.4)	169 (3.3)	
	unknown	2427 (87.0)	483 (94.3)	590 (92.5)	4638 (90.7)	
Bone_M (%)	Blank(s)	1544 (55.3)	378 (73.8)	405 (63.5)	3451 (67.5)	<0.001
	No	1240 (44.4)	134 (26.2)	232 (36.4)	1652 (32.3)	
	Unknown	6 (0.2)	0 (0.0)	1 (0.2)	5 (0.1)	
	Yes	1 (0.0)	0 (0.0)	0 (0.0)	7 (0.1)	
Brain_M (%)	Blank(s)	1544 (55.3)	378 (73.8)	405 (63.5)	3451 (67.5)	<0.001
	No	1242 (44.5)	134 (26.2)	232 (36.4)	1657 (32.4)	
	Unknown	5 (0.2)	0 (0.0)	1 (0.2)	6 (0.1)	
	Yes	0 (0.0)	0 (0.0)	0 (0.0)	1 (0.0)	
Liver_M (%)	Blank(s)	1544 (55.3)	378 (73.8)	405 (63.5)	3451 (67.5)	<0.001
	No	1241 (44.5)	134 (26.2)	229 (35.9)	1656 (32.4)	
	Unknown	5 (0.2)	0 (0.0)	1 (0.2)	5 (0.1)	
	Yes	1 (0.0)	0 (0.0)	3 (0.5)	3 (0.1)	
Lung_M (%)	Blank(s)	1544 (55.3)	378 (73.8)	405 (63.5)	3451 (67.5)	<0.001
	No	1238 (44.4)	134 (26.2)	229 (35.9)	1652 (32.3)	
	Unknown	6 (0.2)	0 (0.0)	1 (0.2)	5 (0.1)	
	Yes	3 (0.1)	0 (0.0)	3 (0.5)	7 (0.1)	
Race (%)	Black	162 (5.8)	57 (11.1)	67 (10.5)	737 (14.4)	<0.001
	Other (American Indian/AK Native, Asian/Pacific Islander)	360 (12.9)	69 (13.5)	80 (12.5)	669 (13.1)	
	Unknown	21 (0.8)	2 (0.4)	4 (0.6)	29 (0.6)	
	White	2248 (80.5)	384 (75.0)	487 (76.3)	3680 (71.9)	

Obviously, negative lymph node was in a comparable large proportion of included cases, ranging from 71.1% to 85.7%. However, for other reasons, most of those retrieved cases did not present full TNM stage information, with 94.9%–97.8% of cases in TX stage, 94.9%–97.6% in NX stage and 94.8%–97.6% in MX stage. Therefore, subdivision analysis based on T stage is not employed in this study. The age, tumor size, surgery status, distribution across four histological sets was statistically significant. Significant distant metastasis lesions (bone/brain/liver/lung) were also found across each histological set (Table 1).

**Fig. 2:**
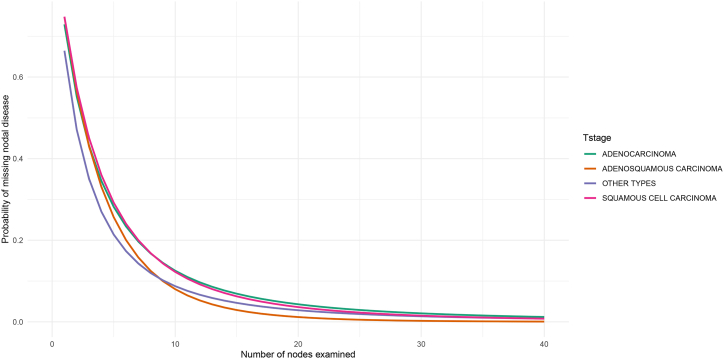
Computation of probability of missing nodal disease. The probability curves of four histological sets, adenocarcinoma, adenosquamous carcinoma, squamous cell carcinoma and other types, were presented with the function of number of examined nodes.

To compute the probability of missing nodal disease over the specific number of examined lymph nodes, a beta-binomial model was used with predefined α and βparameters (Table 2). The probability was then to be presented in four histological types, respectively (Fig. 2).

**Table 2:** Parameters,α/β, is determined by the beta-binomial model used in this study. CI: confidential interval.

Cervical cancer type	α(95% CI)	β(95% CI)
Squamous cell carcinoma	0.17182544 (0.15364983–0.19641145)	2.806309 (2.357267–3.323467)
Adenocarcinoma	0.08012728 (0.06821946–0.09534602)	2.012756 (1.668980–2.532718)
Adenosquamous carcinoma	0.26827863 (0.18419752–0.41277291)	3.456867 (2.122543–5.510791)
Other types	0.12120815 (0.08348291–0.17808531)	1.638166 (1.092888–2.867572)

Apparently, as the number of node examined increased, the false negative probability would reach close to the limit value of 0. However, there may be difference among each histological set in this probability presentation. To reach a comparable false negative probability of 0.05, squamous cell carcinoma patients need at least 17 lymph nodes to be examined, whereas 18 lymph nodes in adenocarcinoma, 12 in adenosquamous carcinoma patients and 14 in other types.

**Fig. 3:**
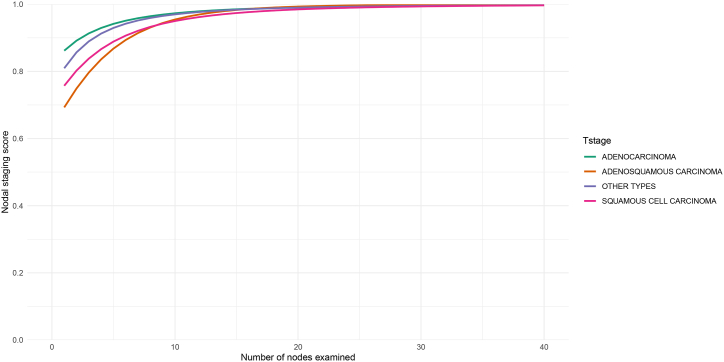
A nodal staging score (NSS) was established to evaluate the difference among each histological set, adenocarcinoma, adenosquamous carcinoma, squamous cell carcinoma and other types in cervical cancer patients.

Next, the NSS was computed based on prevalence value (Table 3). NSS indeed showed the actual status of negative lymph nodes of cervical cancer patients. In squamous cell carcinoma, it only took 2 lymph nodes for NSS to reach 0.8, and 10 lymph nodes to reach 0.95. In adenocarcinoma, all the NSS values were >0.8, with only 6 lymph nodes to reach 0.95. In adenosquamous carcinoma, it took 4 lymph nodes to reach 0.8, and 10 lymph nodes to reach 0.95. In other types, it took 7 lymph nodes to reach 0.95 (Fig. 3). Different histological types dictate various NSS threshold, which may facilitate future surgical lymph node dissection requirement in a histologic-specific manner.

**Table 3:** Both apparent and adjusted prevalence of each type of cervical cancer.

Cervical cancer type	Apparent prevalence (%)	Adjusted prevalence (%)
Squamous cell carcinoma	0.2297165	0.3000740
Adenocarcinoma	0.1426012	0.1803439
Adenosquamous carcinoma	0.2890625	0.3724601
Other types	0.2037618	0.2617049

**Fig. 4:**
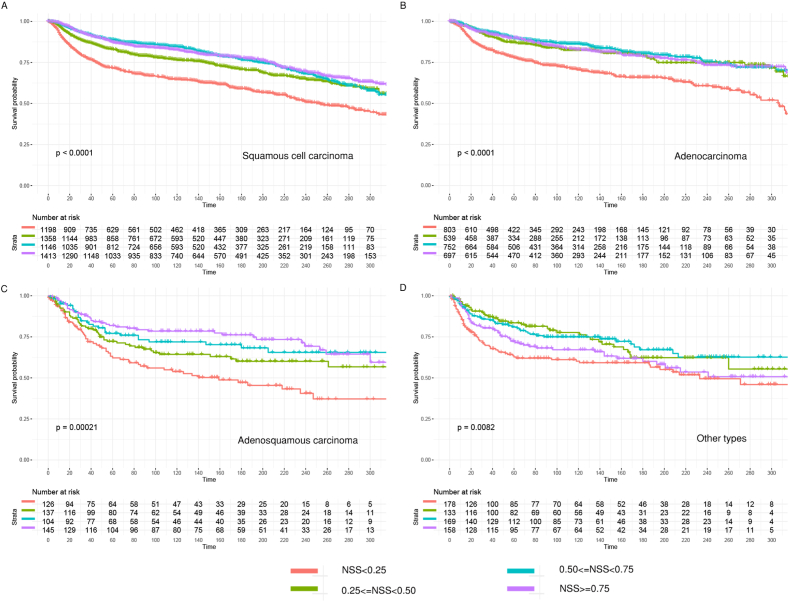
Kaplan-Meier overall survival analysis of different histological sets in cervical cancer patients. (A) The quartile NSS cutoffs in squamous cell carcinoma were 0.9500764, 0.9791477, 0.9894570; (B) The quartile NSS cutoffs in adenocarcinoma were 0.9763910, 0.9877181, 0.9931550; (C) The quartile NSS cutoffs in adenosquamous carcinoma were 0.9629919, 0.9882775, 0.9963854; (D) The quartile NSS cutoff in other types were 0.9651469, 0.9853985, 0.9921761.

To further evaluate the prognostic values of NSS based subsets, the NSS value quartile of each histological sets were determined. Given diverse NSS indicates different possibility of missing lymph node disease, four groups based on NSS quartile (group with NSS<0.25, 0.25 ≤ NSS<0.5, 0.5 ≤ NSS<0.75, 0.75 ≤ NSS) were analyzed. NSS based quartile subsets served to be a significant prognostic tool in cervical cancer patients. In fact, NSS<0.25 group remarkably showed the worst clinical outcome in squamous cell carcinoma, adenocarcinoma, adenosquamous carcinoma and other types (Fig. 4A–D). Consistently, low NSS represented high possibility of missing lymph nodes disease status, which in turn leads to potential higher risk of recurrence or relapse.

## 4 Discussion

Histological types of cervical cancer are commonly used to identify potential therapeutic subsets or prognostic prediction [4,5]. Our study is the first to apply NSS in cervical cancer with histological features. Within 9056 patients in this study, in order to reduce the false negative probability below 0.05, the required number of examined lymph nodes was calculated to be 17 in squamous cell carcinoma, 18 lymph nodes in adenocarcinoma, 12 in adenosquamous carcinoma patients and 14 in other types. This finding is of significance in both clinical and pathological senses, given negative lymph node was in a large proportion in each histological set, ranging from 71.1% to 85.7%. For instance, if an adenocarcinoma cervical cancer patient received curative lymphadenectomy with 20 lymph nodes total negative, the probability of finding a false negative lymph node is < 0.05.

In fact, false negative probability of lymph nodes, or nodal disease, is a critical risk for tumor recurrence across various malignancies. Both surgeons and pathologist are the primary determinants for precise diagnosis of adequate nodal status. Nonetheless, staging error or miscalculation remains. NSS offers a statistical approach for minimum requirement of lymph node dissection, and a reliable tool to correlate with tumor histology in this scenario.

Noteworthy, previous NSS studies, such as Wu et al., assessed the NSS in different tumor stages [12,13]. However, most of recruited individuals in this study do not show complete TNM stage information. TX was found in 94.9%–97.8% of cases. Therefore, it is not feasible to apply tumor stage into present study. However, distinct tumor histology types have been introduced for the first time. Of note, both nodal staging and tumor histology are the major determinant factors for postoperative therapeutic course. Statistically expedition on both factors remains sparse. Conventional analysis may prefer classification tree, institutional retrospective data, or logistic regression for prediction. Thereby, by employment of diagnostic test statistical standards, this study not only focuses on the minimum number of lymph nodes to be examined, but also highlights the value of specific histologic types.

There are several advantages in NSS. First, the application of NSS in node-negative individual could directly ensure the true negative result of nodal disease in each patient with quantitative description, contributing to further clinical counseling and therapeutic choice. Second, the effect of histological types on clinical management is yet to be fully acknowledged. A histology-specific NSS results indeed naturally provide accurate value of number of examined/positive lymph nodes.

There are many histological types that are categorized into other types, including carcinosarcoma, lymphoma, germ cell tumor, Mullerian tumor, mast cell tumors and other types with very rare cases. Carcinosarcomas in cervical cancer are very rare, but show very poor prognosis, with 5-year survival at stage IV almost 0% [[14], [15], [16]]. Lymphoma is another rare type in cervical cancer [17]. It remains difficult to establish a well confirmed diagnosis in lymphoma of uterine cervix [17,18]. Such classification in this study enables a comparable sample size and clear demonstration.

Limitations of this study include I. original data resource is from the SEER, United States-based population database, may not fully characterize the population-specific lymph node information; II. the nature of this study is distinct from randomized clinical trials or cohort study; III. although numerous types of histology, including sarcoma and lymphoma, in cervical cancer have been assigned into other types, we believe it is still of huge clinical value to explore the NSS model in those types of cervical cancer, individually.

## 5 Conclusion

NSS tool enables adequate nodal staging of cervical cancer with significant prognostic value. Exact number of lymph nodes required for surgery in cervical cancer is specified based on histologic type.

## Funding

This work was supported by Science and Technology Commission of Shanghai Municipalicy (grant no. 11ZR1407700) of Yan Huang and 10.13039/100014717National Natural Science Foundation of China (grant no. 81202051) of Yan Huang.

## Ethical approval

This article does not contain any studies with human participants or animals performed by any of the authors.

## Informed consent

No informed consent was needed in the study.

## Consent for publication

Not applicable.

## Data availability statement

The datasets supporting the conclusion of this article are included within the article.

Has data associated with your study been deposited into a publicly available repository? No.

## Additional information

No additional information is available for this paper.

## CRediT authorship contribution statement

**Rui Jiang:** Writing – review & editing, Writing – original draft, Visualization, Validation, Software, Resources, Methodology, Investigation, Data curation, Conceptualization. **Xiaoqi Li:** Writing – review & editing, Writing – original draft, Validation, Resources, Methodology, Investigation, Data curation, Conceptualization. **Siyu Cao:** Writing – review & editing, Writing – original draft, Visualization, Validation, Software, Resources, Methodology, Investigation. **Yong Wu:** Writing – review & editing, Writing – original draft, Visualization, Validation, Resources, Conceptualization. **Wei Zhang:** Writing – original draft, Visualization, Validation, Methodology, Investigation, Formal analysis, Data curation. **Yan Huang:** Writing – review & editing, Writing – original draft, Visualization, Validation, Supervision, Project administration, Methodology, Investigation, Funding acquisition, Formal analysis, Data curation, Conceptualization.

## Declaration of competing interest

The authors declare that they have no known competing financial interests or personal relationships that could have appeared to influence the work reported in this paper.
